# Human alpha-synuclein overexpressing MBP29 mice mimic functional and structural hallmarks of the cerebellar subtype of multiple system atrophy

**DOI:** 10.1186/s40478-021-01166-x

**Published:** 2021-04-14

**Authors:** Lisa Mészáros, Markus J. Riemenschneider, Heiko Gassner, Franz Marxreiter, Stephan von Hörsten, Alana Hoffmann, Jürgen Winkler

**Affiliations:** 1 Department of Molecular Neurology, University Hospital Erlangen, Friedrich-Alexander-Universität Erlangen-Nürnberg, 91054 Erlangen, Germany; 2 Department of Neuropathology, Regensburg University Hospital, 93053 Regensburg, Germany; 3 Experimental Therapy, University Hospital Erlangen, Friedrich-Alexander-Universität Erlangen-Nürnberg, 91054 Erlangen, Germany

**Keywords:** Multiple system atrophy, Cerebellar pathology, Cerebellar ataxia, Gait analysis

## Abstract

Multiple system atrophy (MSA) is a rare, but fatal atypical parkinsonian disorder. The prototypical pathological hallmark are oligodendroglial cytoplasmic inclusions (GCIs) containing alpha-synuclein (α-syn). Currently, two MSA phenotypes are classified: the parkinsonian (MSA-P) and the cerebellar subtype (MSA-C), clinically characterized by predominant parkinsonism or cerebellar ataxia, respectively. Previous studies have shown that the transgenic MSA mouse model overexpressing human α-syn controlled by the oligodendroglial myelin basic protein (MBP) promoter (MBP29-hα-syn mice) mirrors crucial characteristics of the MSA-P subtype. However, it remains elusive, whether this model recapitulates important features of the MSA-C-related phenotype. First, we examined MSA-C-associated cerebellar pathology using human *post-mortem* tissue of MSA-C patients and controls. We observed the prototypical GCI pathology and a preserved number of oligodendrocytes in the cerebellar white matter (cbw) accompanied by severe myelin deficit, microgliosis, and a profound loss of Purkinje cells. Secondly, we phenotypically characterized MBP29-hα-syn mice using a dual approach: structural analysis of the hindbrain and functional assessment of gait. Matching the neuropathological features of MSA-C, GCI pathology within the cbw of MBP29-hα-syn mice was accompanied by a severe myelin deficit despite an increased number of oligodendrocytes and a high number of myeloid cells even at an early disease stage. Intriguingly, MBP29-hα-syn mice developed a significant loss of Purkinje cells at a more advanced disease stage. Catwalk XT gait analysis revealed decreased walking speed, increased stride length and width between hind paws. In addition, less dual diagonal support was observed toward more dual lateral and three paw support. Taken together, this wide-based and unsteady gait reflects cerebellar ataxia presumably linked to the cerebellar pathology in MBP29-hα-syn mice. In conclusion, the present study strongly supports the notion that the MBP29-hα-syn mouse model mimics important characteristics of the MSA-C subtype providing a powerful preclinical tool for evaluating future interventional strategies.

**Supplementary Information:**

The online version contains supplementary material available at 10.1186/s40478-021-01166-x.

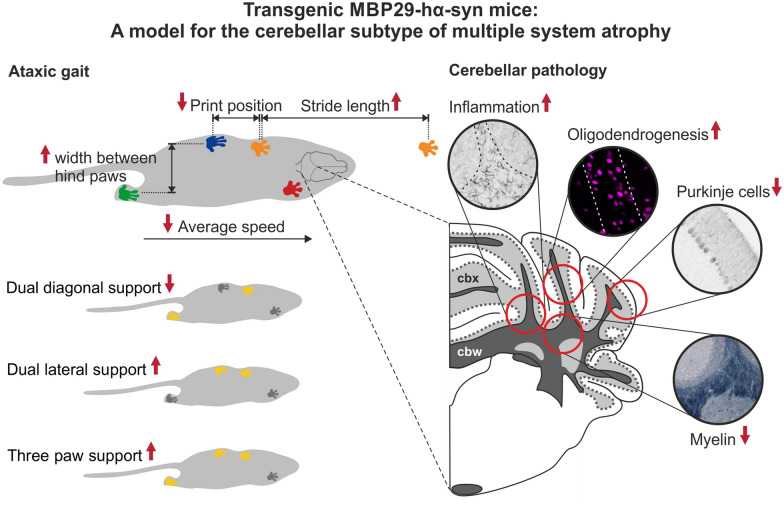

## Introduction

Multiple system atrophy (MSA) is an orphan, sporadic, and rapidly progressive neurodegenerative disease with equal gender distribution and a mean life expectancy of 6.2–10 years after diagnosis [1–4]. Besides a severe autonomic failure as a mandatory feature for diagnosis, MSA is categorized in two distinct motor subtypes. The parkinsonian subtype (MSA-P) shows predominantly L-Dopa non-responsive parkinsonism including bradykinesia, rigidity, and postural instability, and is associated with striatonigral degeneration (SND). The cerebellar subtype (MSA-C) is characterized by cerebellar ataxia of the limbs with signs of a severe olivopontocerebellar atrophy (OPCA) [3, 5–7]. Interestingly, MSA-P is more frequently observed in Western regions (70–80%), whereas MSA-C is more common in Asian populations (67–84%) indicating unknown effects of ethnicity or environment [1].

Neuropathologically, MSA-C is characterized by oligodendroglial cytoplasmic inclusions (GCIs) of alpha-synuclein (α-syn) initially within the pontine cerebellar projections and cerebellar white matter (cbw) followed by accumulations within the pyramidal and extrapyramidal white matter [3, 8, 9]. The main post-translational modification of α-syn in GCIs is phosphorylated α-syn at the Ser129 residue [10]. To date, it is still controversial whether GCIs lead to oligodendroglial degeneration [11–13]. Nevertheless, there is strong evidence that α-syn accumulation results in an oligodendroglial dysfunction leading to reduced levels of myelin [12, 14]. Indeed, myelin loss within the pons and cerebellum correlates with the level of GCI pathology and aggravates with disease duration [8]. Moreover, oligodendroglial dysfunction is characterized by enlargement of the oligodendroglial cytoplasm with relocalization of tubulin polymerization-promoting protein p25α (TPPP/p25α) from the nucleus to the cytoplasm [15]. Additionally, neuroinflammation is an important pathological feature of MSA-C. Ishizawa and colleagues described an increased microglial burden within the cbw of MSA (MSA-P and MSA-C) patients correlating with the number of GCIs [9]. Associated with GCI burden, demyelination, as well as neuroinflammation, neuronal cell loss in inferior olives, pontine nuclei and, in particular, degeneration of Purkinje cells are other important and progressive cerebellar hallmarks of MSA-C [8, 16–18]. Notably, this neuronal loss is linked to a severe limb and gait ataxia in MSA-C patients [19].

Currently, there is no disease-modifying therapy available for MSA. Even symptomatic treatment is still limited, in particular for cerebellar ataxia [20]. However, the rapid and severe progression of MSA-C as well as its orphan disease status makes MSA-C attractive for advanced drug development. Thus, robust MSA-C mouse models are an important prerequisite for an effective pharmaceutical development.

In previous studies we have characterized the MSA-P-related neuropathology in the forebrain of human α-syn-overexpressing mice. In these mice, human α-syn expression is controlled by the murine oligodendroglial myelin basic protein promoter (MBP29-hα-syn mice) [21–25]. Although Shults and colleagues noticed α-syn expression in the cerebellum of this mouse model, the cerebellar pathology and functional consequences remain largely elusive [25]. We therefore hypothesized that MBP29-hα-syn mice mirror important features of the MSA-C-associated cerebellar pathology leading to behavioral changes in cerebellum-related motor functions.

In order to revisit crucial aspects of MSA-C, we first aimed to examine MSA-C-relevant cerebellar pathology using human *post-mortem* cerebellar tissue of MSA-C patients and controls. Besides α-syn pathology we focused on the level of myelin, the number of oligodendrocytes, the neuroinflammatory pattern, and the number of Purkinje cells. Furthermore, we characterized the cerebellum-related pathology in the present transgenic mice at a prodromal stage and a more advanced disease stage to shed more light on disease progression. Finally, we assessed changes in gait pattern using automated gait analysis. The Catwalk XT system, which has already been established as an ideal tool for assessing ataxia, was used to characterize functional deficits [26–29].

## Material and methods

### Human *post-mortem* tissue

Human *post-mortem* samples of five MSA-C patients and five age- and gender-matched controls (Table 1) were obtained from the Netherlands Brain Bank (NBB), Netherlands Institute for Neuroscience, Amsterdam (open access: http://www.brainbank.nl) [30, 31]. All MSA cases were clinically diagnosed and neuropathologically confirmed according to the second consensus criteria [5]. Importantly, all MSA patients developed cerebellar ataxia as one important clinical manifestation of MSA-C. Paraffin-embedded tissues of the cerebellum were sectioned into 4 µm thick sections. After deparaffinization, three sections of each individual were stained for Sudan Black (15 min incubation of 1% Sudan Black solution) or haematoxylin and eosin (H&E, 10 min incubation of haematoxylin followed by eosin for 30 s). Immunohistochemical stainings were performed after additional epitope retrieval (citrate buffer, 10 mmol/L, pH 6.0) using the following primary antibodies: CD68 (1:600, KP1, M0814, Dako), oligodendrocyte transcription factor 2 (OLIG2, 1:200, ab109186, Abcam), and α-syn (1:500, AB5038P, Merck Millipore). Afterwards primary antibodies were labeled using the OptiView DAB IHC Detection Kit (760-700, Ventana, Roche) or for co-staining the EnVision G/2 Doublestain System, Rabbit/Mouse (DAB+/Permanent Red, K5361, Dako). Nuclei were counterstained using haematoxylin. For all images and analyses an Imager.M2 microscope (Zeiss) was used in conjunction with the Stereo Investigator software (MBF Bioscience). Sudan Black staining intensity of the subcortical cbw was measured by the mean gray value normalized to the cerebellar cortex (cbx) using ImageJ software. H&E staining was used to quantify the number of Purkinje cells. CD68^+^ cells were quantified within the cbw and cbx using six images per slice combined with the cell counter plugin of the ImageJ software. α-syn^+^ and OLIG2^+^ cells were quantified using the Stereo Investigator software (MBF Bioscience).

**Table 1:** Demographic and clinical characteristics of MSA‐C patients and controls

Diagnosis	Gender	Age (years)	*Post-mortem* delay (min)	Disease duration (months)	Brain weight (g)	Cause of death
Control	F	75	550	-	1305	Euthanasia
Control	F	60	330	-	1215	Euthanasia
Control	F	55	450	-	1260	Euthanasia
Control	F	70	375	-	1188	Pulmonary carcinoma
Control	M	72	260	-	1385	Endocarditis
	4F/1 M	66 ± 8	398 ± 111	-	1291 ± 63	
MSA-C	F	52	335	50	1095	Euthanasia
MSA-C	F	70	405	69	1178	Euthanasia
MSA-C	F	69	480	46	1265	Euthanasia
MSA-C	F	62	335	48	1050	Euthanasia
MSA-C	M	70	415	46	1325	Euthanasia
	4F/1 M	65 ± 7	394 ± 55	52 ± 9	1183 ± 102	

*Post‐mortem* cerebellar tissue (n = 5 per group) were obtained from the Netherlands Brain Bank (NBB), Netherlands Institute for Neuroscience, Amsterdam (open access: http://www.brainbank.nl). MSA‐C patients and controls do not differ in gender (F for female, M for male), age, and *post‐mortem* delay (Pearson's chi-squared test, Mann–Whitney test: p > 0.05)

### Animals

**Fig. 1:**
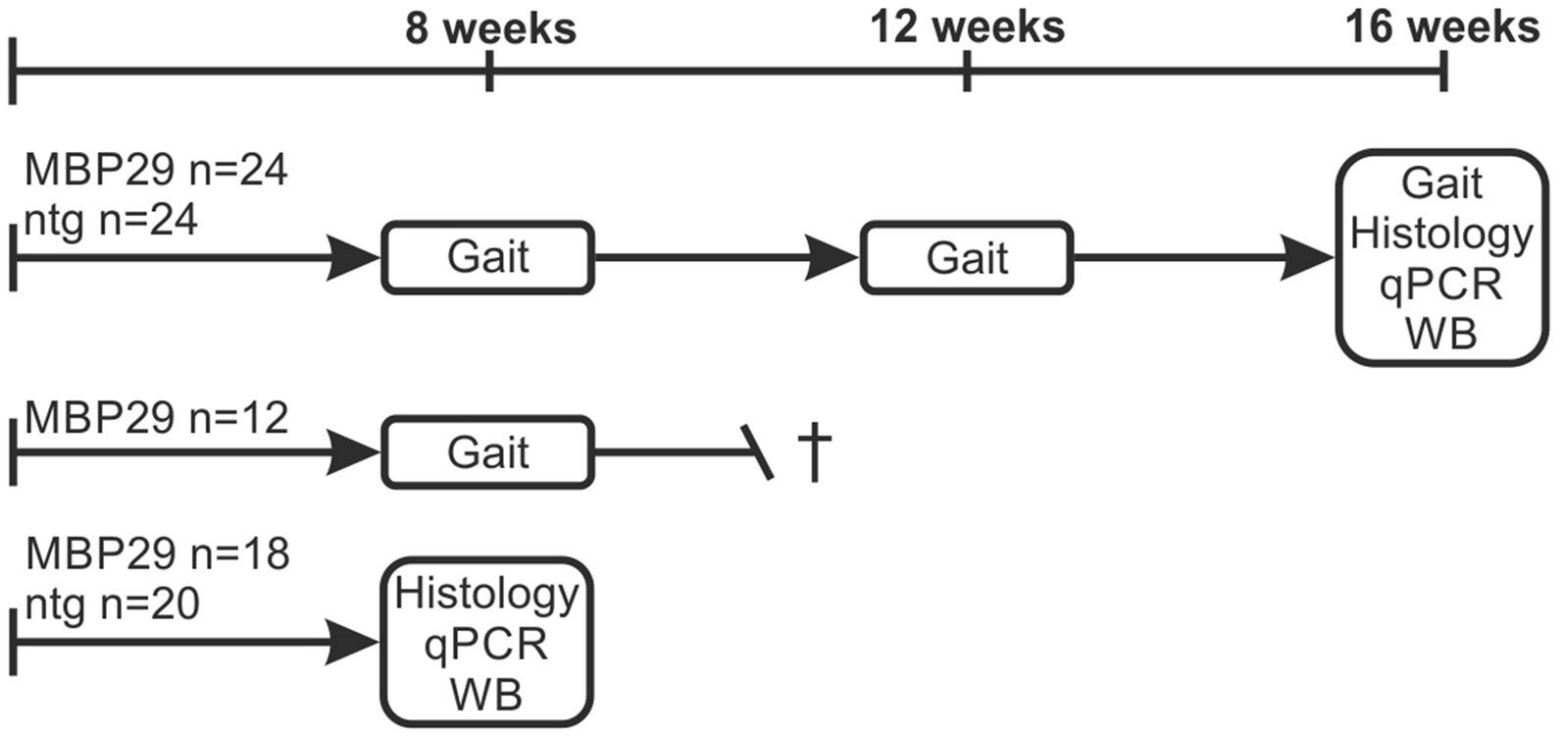
Experimental paradigm. For longitudinal gait analysis, 24 MBP29-hα-syn (MBP29) mice that completed gait analysis were compared to 24 control mice (ntg). For the subgroup gait analysis at an age of 8 weeks, 24 MBP29-hα-syn mice were compared to 12 MBP29-hα-syn mice that were not able to be assessed completely. Following gait analysis, the 16-week-old mice and an additional cohort of 8-week-old mice (n = 18 MBP29, n = 20 ntg) were transcardially perfused and cerebellar tissue was used for histological, mRNA (quantitative real-time PCR, qPCR), and protein analysis (Western Blot, WB)

Transgenic MBP29-hα-syn mice overexpressing human α-syn under the control of the murine MBP promoter were compared to non-transgenic littermates as controls at an age of 8, 12, and 16 weeks using a longitudinal behavioral design for gait analysis (Fig. 1). MBP29-hα-syn mice show a severe neurological phenotype including tremor and ataxia at an advanced stage and die prematurely [22, 25]. Following gait analysis, the 16-week-old mice and an additional cohort of 8-week-old mice were transcardially perfused using 0.9% sodium chloride solution according to the European and National Institute of Health guidelines for humane treatment of animals. Afterwards, brains were transferred into 4% paraformaldehyde for 24 h and subsequently transferred into 30% sucrose. Cerebellar hemispheres were coronally sectioned into 20 µm thick slices using a sliding microtome and kept in cryoprotection solution (25% 0.2 mol/L phosphate buffer) at − 20 °C until staining. For protein and RNA analysis, the cerebella were microdissected and stored at − 80 °C until tissue homogenization.

### Gait analysis

The Catwalk XT 10.0 gait analysis system (Noldus Information Technology, Wageningen, Netherlands) was used to analyze changes in gait parameters associated with cerebellar dysfunction in 36 MBP29-hα-syn mice (15 male, 21 female) as well as in 24 control mice (12 male, 12 female) at an age of 8, 12, and 16 weeks (Fig. 1) according to a previously described protocol [29]. In brief, animals crossed a glass walkway while a camera was recording the paw prints from below. After presetting the camera gain (25.0 dB) and intensity threshold (0.1), up to 30 runs were recorded for each animal in order to obtain a representative number of completed runs per animal. A run was defined as completed when the animal walked continuously without stopping along the walkway. The gait parameters obtained from completed runs were semi-automatically scored by the Catwalk XT software and the means of the runs were analyzed. We thereby focused on gait parameters previously identified as reliable indicators for ataxia (Additional file 1. Table S1) [26–28]. For the longitudinal analysis, 24 MBP29-hα-syn mice (10 male, 14 female) that completed gait analysis were compared to 24 control mice. For the subgroup analysis at an age of 8 weeks, 24 MBP29-hα-syn mice were compared to 12 MBP29-hα-syn mice (5 male, 7 female) that did not complete the last time point at 16 weeks due to the progressive nature of the disease course. Behavioral characterization was approved by the local Government of Bavaria, Germany (#55.2-DMS 2532-2-218).

### Diaminobenzidin (DAB) staining

For antigen retrieval, three free-floating cerebellar sections from each of six transgenic and six control mice (at an age of 8 and 16 weeks) were pretreated using citrate buffer for 30 min at 80 °C followed by an incubation at room temperature (RT) for 30 min. To inhibit endogenous peroxidase activity, 0.6% H_2_O_2_ was used for 30 min at RT. After blocking for 1 h at RT with blocking solution (3% donkey serum, 0.3% Triton-X100), sections were stained for 24 h at 4 °C using primary antibodies (diluted in blocking solution) either against ionized calcium-binding adapter molecule 1 (IBA1, 1:500, 019-19741, Wako) or calbindin D28k (CALB, 1:200, 300, Swant). Afterwards, a biotin-conjugated antibody was used to label the primary antibody (donkey anti-rabbit biotin, 1:1000, 711-065-152, Dianova or donkey anti-mouse biotin, 1:1000, 715-065-151, Dianova). Following incubation with avidin-peroxidase-complex (Vectastain Elite ABC HRP Kit, PK-6100, Vector Laboratories) for 30 min at RT, DAB Peroxidase (HRP) Substrate Kit with nickel (SK-4100, Vector Laboratories) was used for visualization. Cell numbers, the area of the regions of interest (ROI), and the length of the Purkinje cell monolayer were measured using an Imager.M2 microscope (Zeiss) supported by the Stereo Investigator software (MBF Bioscience).

### Immunofluorescence staining

After pretreatment with citrate buffer and blocking solution (see above) for 1 h at RT, three free-floating sections per animal (n = 6 per genotype and age) were stained with the following primary antibodies over-night at 4 °C: OLIG2 (1:500, AB9610, Merck Millipore), platelet-derived growth factor receptor alpha (PDGFRα, 1:250, AF1062, R&D Systems), α-syn (1:200, 15G7, ALX-804-258, Enzo Life Sciences), phosphorylated α-syn at Ser129 (pS129-α-syn, 1:500, ab51253, Abcam), and TPPP/p25α (1:200, ab92305, Abcam). For fluorescence staining, the following secondary antibodies were incubated for 1 h at RT: Alexa Fluor 647 donkey anti-rabbit (1:1000, 711-605-152, Dianova), Alexa Fluor 568 donkey anti-goat (1:1000, A11057, Life Technologies), and Alexa Fluor 488 donkey anti-rat (1:1000, A21208, Life Technologies). Nuclei were counterstained with DAPI (1:10,000, D8417, Sigma) for 10 min. To analyze the cell density in the ROI, three Z stack images were taken in each region using the fluorescence Observer microscope (Zeiss) in conjunction with the ZEN blue software. Cell density and the area of OLIG2^+^ cells were quantified using the cell counter plugin and the analyze particles plugin of the ImageJ software, respectively.

### Myelin staining

A modified version of the Heidenhain Woelcke staining was performed using five free-floating sections per animal (n = 5–6 per genotype and time point) [23, 32, 33]. Sections were incubated in 2.5% iron alum solution for 30 min at RT. Following short rinses with deionized water, sections were transferred into a freshly prepared staining solution (10% haematoxylin, 1% lithium carbonate) for 20 min at RT and rinsed again in deionized water. Overview images were taken with identical exposure time using an Imager.M2 microscope (Zeiss) and Stereo Investigator software (BMF Bioscience). To quantify the staining intensity of the ROI, the mean gray value of 8-bit images was analyzed using the ImageJ software and normalized to the molecular layer of the cbx.

### Quantitative real-time PCR (qPCR)

For RNA analysis, cerebellar tissue (n = 5 per genotype and time point) was homogenized using QIAzol Lysis Reagent (79306, Qiagen). After phase separation using chloroform and following centrifugation, RNA was purified using an RNeasy mini kit (74136, Qiagen). RNA concentration was measured using Nano-Drop technology (PeqLab). GoScript™ Reverse Transcription System (A5004, Promega) was applied for cDNA production. To quantify gene transcription, qPCRs were performed on a LightCycler 480 (Roche) using the SSo Fast EvaGreen Supermix (1725205, Bio-Rad Laboratories) and following primers: *Calb* (forward TTG GCT CAC GTC TTA CCC AC, reverse TGC ACT GGT AGT AAC CTG GC), *18S rRNA* (forward GGA CCA GAG CGA AAG CAT TTG, reverse GCC AGT CGG CAT CGT TTA TG), and β*-actin* (forward GCC TTC CTT GGG TAT GGA A, reverse CAG CTC AGT AAC AGT CCG CC).

### Western blot

Cerebella of transgenic and control mice (n = 5 per genotype and time point) were homogenized in radioimmunoprecipitation assay (RIPA) buffer (50 mM Tris–HCl, pH 8.0, 150 mM NaCl, 2 mM EDTA, 1% v/v Nonidet P-40 (Roche), 0.5% v/v Na-deoxycholate (Roth), 0.1% w/v SDS (Applichem), complemented with protease inhibitor (Roche) and phosphatase inhibitor (Roche)). Protein concentration was measured using bicinchoninic acid (BCA) assay (Pierce BCA Protein Assay Kit, Thermo Scientific) and ClarioStar Microplate Reader (562 nm). Total protein (10–30 µg) was separated on 4–12% Bis–Tris gels (NP0322BOX, Invitrogen) and blotted onto a polyvinylidene difluoride membrane for fluorescence applications (PVDF-FL, IPFL 00010, Millipore). Membranes were blocked (1% w/v bovine serum albumin) for 2 h at RT followed by incubation over-night at 4 °C using the following primary antibodies: myelin basic protein (MBP, 1:500, MCA409S, Bio-Rad Laboratories), proteolipid protein 1 (PLP, 1:1000, ab28486, Abcam), β-actin (β-act, 1:500, AB8226, Abcam), and CALB (1:500, 13176P, Cell Signaling Technology). Afterwards, the following fluorescent secondary antibodies were applied for 1 h at RT: Alexa Fluor 647 donkey anti-rabbit (1:1000, 711-605-152, Dianova), Alexa Fluor 488 donkey anti-rat (1:1000, A21208, Life Technologies), and Alexa Fluor 488 donkey anti-mouse (1:1000, A21202, Life Technologies). Protein bands were visualized and analyzed using FusionFX7 (Peqlab) and the Bio1D software (Vilber Lourmat), respectively.

### Statistical analyses

Data were analyzed using IBM SPSS Statistics software and were visualized using GraphPad Prism and CorelDRAW X6 software. Pearson's chi-squared and Mann–Whitney test revealed no sex- and age-related differences between MSA patients and controls nor differences in group size and sex between MBP29-hα-syn mice and control mice. To determine normal distribution of parameters, the Shapiro–Wilk test was used. Even though most of the parameters were normally distributed, the Mann–Whitney test (non-parametric, unpaired) was chosen for all analyses due to low sample sizes. To evaluate disease-related progression in gait parameters of MBP29-hα-syn mice at 8 and 16 weeks of age, the Wilcoxon signed-rank test (non-parametric, paired) was performed. All parameters expressed in length units were normalized to the mean bodyweight of all mice at each time point to avoid confounding effects of bodyweight on gait parameters due to mixed-sex cohorts, weight gain during gait analysis, and bodyweight differences between sex, transgenic mice and controls (Additional file 1. Table S1). Unless indicated differently, all analyses are expressed as mean + standard deviation. Effect size r was calculated (according to Rosenthal 1991 [34]) using the z-score (obtained from Mann–Whitney test). P values ≤ 0.05 were considered statistically significant.

## Results

### Severe pathology in the cerebellar white matter of MSA-C patients

**Fig. 2:**
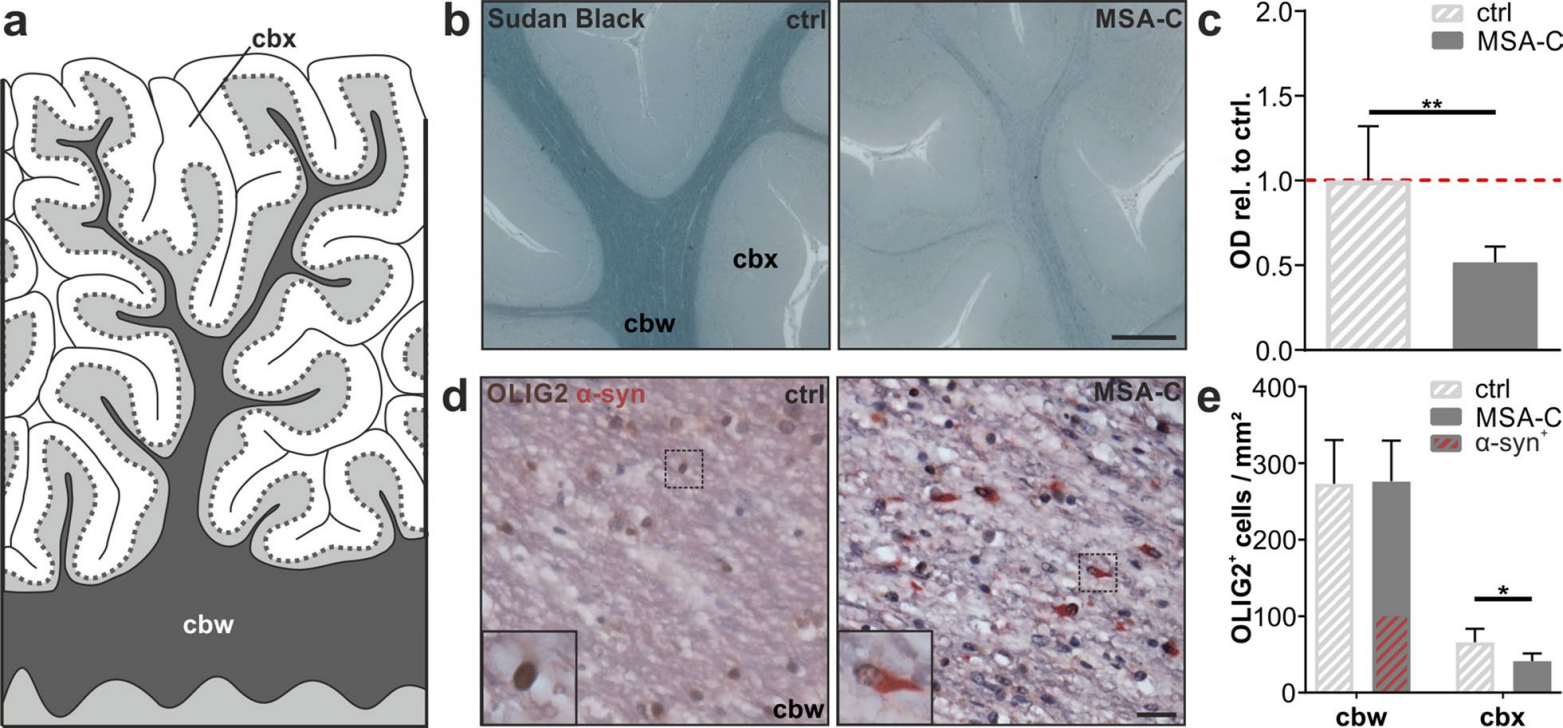
Myelin loss and widespread GCI pathology, but preserved number of oligodendrocytes in the cerebellar white matter of MSA-C patients. **a** Graphical overview illustrating regions of interest within the human cerebellum including the cerebellar white matter (cbw) and cerebellar cortex (cbx). **b** Sudan Black staining of the cerebellum of MSA-C patients shows a severe myelin deficit compared to controls; scale bar: 500 µm. **c** Quantification of myelin content measured by the optical density (OD) of Sudan Black staining in the subcortical white matter normalized to the molecular layer within the cbx; n = 5. **d** Co-staining of OLIG2 (brown) and α-syn (red) within the cbw; nuclei were counterstained using haematoxylin (blue); scale bar: 20 µm. **e** Note the preserved number of OLIG2^+^ cells in the cbw of MSA-C patients compared to controls (n = 5). 36% of OLIG2^+^ cells show α-syn accumulations of the cbw in MSA-C patients. Inserts show OLIG2^+^ oligodendrocytes. Data represent mean + standard deviation. Statistical analyses were performed using Mann–Whitney test; *p ≤ 0.05, **p ≤ 0.01

Neuropathological examination of MSA-C related pathology within the cerebellum was performed using *post-mortem* cerebellar samples of five MSA-C patients compared to five age- and gender-matched controls (Fig. 2, Table 1). One important hallmark of MSA-C is a severe myelin deficit. To examine the level of myelin in the MSA-C cohort, we performed a Sudan Black staining. This staining visualizes lipids such as phospholipids, sterols, and neutral triglycerides, which represent the most important biochemical components of myelin (Fig. 2b). When evaluating the staining intensity of the subcortical white matter of the cerebellum, we observed a profound loss of myelin by 48% (*p* = 0.008, Fig. 2c). A severe myelin deficit accompanied by a preserved number of immature oligodendrocytes has been reported previously [13, 21]. Thus, we conducted a co-staining of OLIG2 as an oligodendrocyte-specific marker in combination with α-syn in order to identify both the number of oligodendrocytes and the density of oligodendroglial cytoplasmic inclusions containing α-syn (Fig. 2d). We observed no differences in the number of oligodendrocytes in the cbw, however a decrease in the cbx of MSA-C patients (Fig. 2e). More than a third of OLIG2^+^ oligodendrocytes (36%) show α-syn accumulations in the cbw of MSA-C patients (Fig. 2e), while no GCIs were identified in the cbx of MSA-C patients (Additional file 1. Fig. S1).

**Fig. 3:**
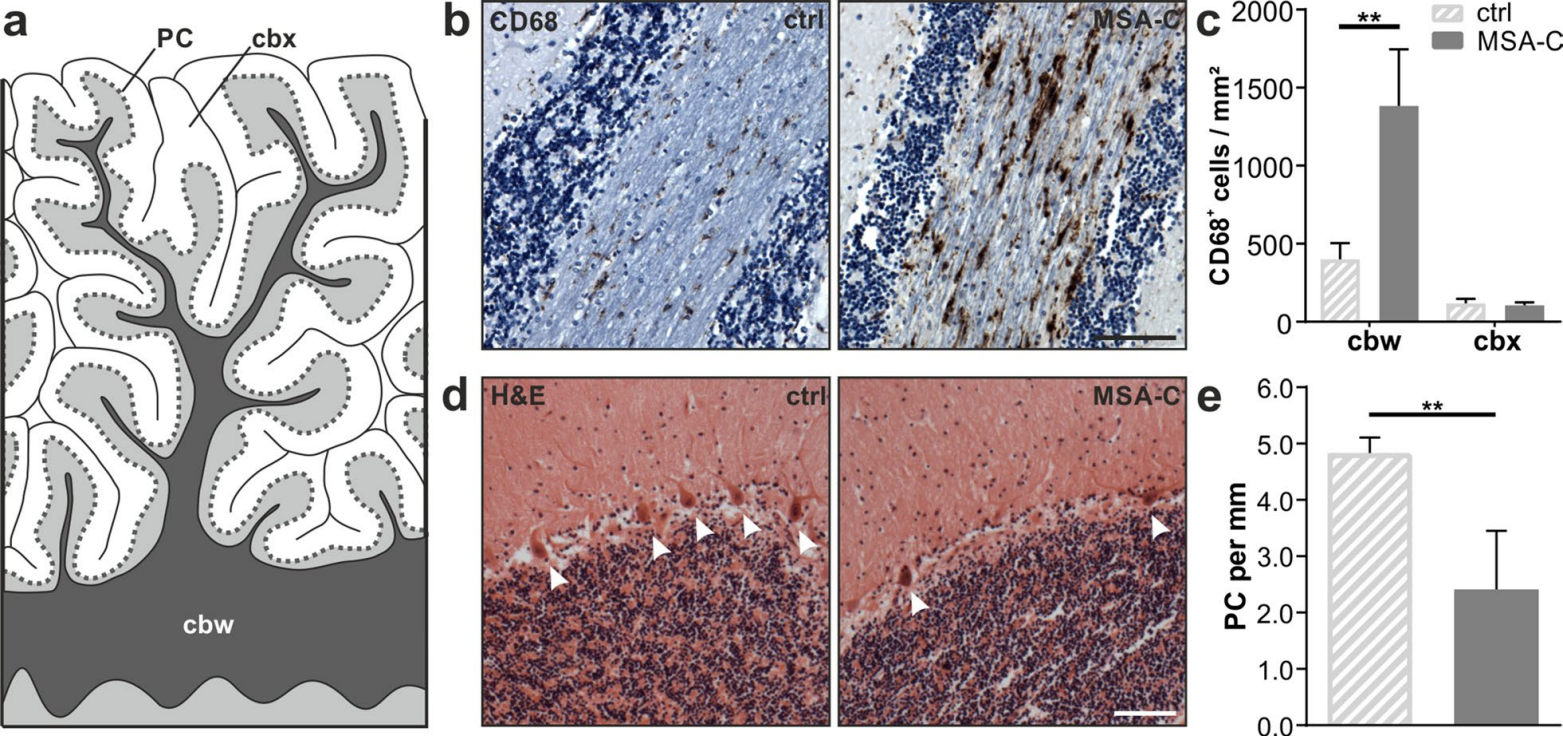
Inflammation within the cerebellar white matter is accompanied by loss of Purkinje cells in MSA-C. **a** Graphical overview illustrating regions of interest within the human cerebellum including the cerebellar white matter (cbw), cerebellar cortex (cbx), and Purkinje cell (PC) monolayer. **b** Cellular expression of CD68; nuclei were counterstained using haematoxylin; scale bar: 100 µm. **c** Quantification of CD68^+^ cells reveals increased microgliosis in the cbw of MSA-C patients (n = 5). **d**, **e** H&E staining of the cerebellum indicates significant loss of Purkinje cells by 50% (white arrow heads) in MSA-C patients compared to controls (n = 5); scale bar: 100 µm. Data represent mean + standard deviation. Statistical analyses were performed using Mann–Whitney test; **p ≤ 0.01

To assess the neuroinflammatory response in cerebellar tissue, we used CD68 as marker for activated myeloid cells (Fig. 3b). We observed an increased density of CD68^+^ cells in the cbw of MSA-C patients compared to controls (3.5-fold, *p* = 0.008, Fig. 3c). Interestingly, there was no difference in the number of CD68^+^ cells in the cbx between MSA-C patients and controls suggesting a severe immune response restricted to the cbw in MSA-C.

Standardized H&E staining was used to quantify the number of Purkinje cells as characteristic inhibitory neurons innervating the cbx and regulating motor coordination and balance. Here, we observed a reduced number of Purkinje cells by 50% in MSA-C compared to controls (*p* = 0.008, Fig. 3d, e). Taken together, our human *post-mortem* data illustrate disease-relevant cerebellar neuropathology of MSA-C patients and thus set the basis for a detailed comparison to the cerebellar pathology of MBP29-hα-syn mice.

### *Cerebellar phenotype of MBP29*-hα-syn *mice resembles MSA-C pathology*

Previously, we and others have demonstrated that MBP29-hα-syn mice display neuropathological characteristics observed in MSA-P patients [21, 22, 25]. In order to investigate, whether this mouse model also represents a promising preclinical tool for the cerebellar subtype of MSA, we first aimed to characterize the cerebellar pathology at two time points, a prodromal disease stage without showing an obvious motor phenotype (8 weeks) and a more advanced disease stage (16 weeks).

**Fig. 4:**
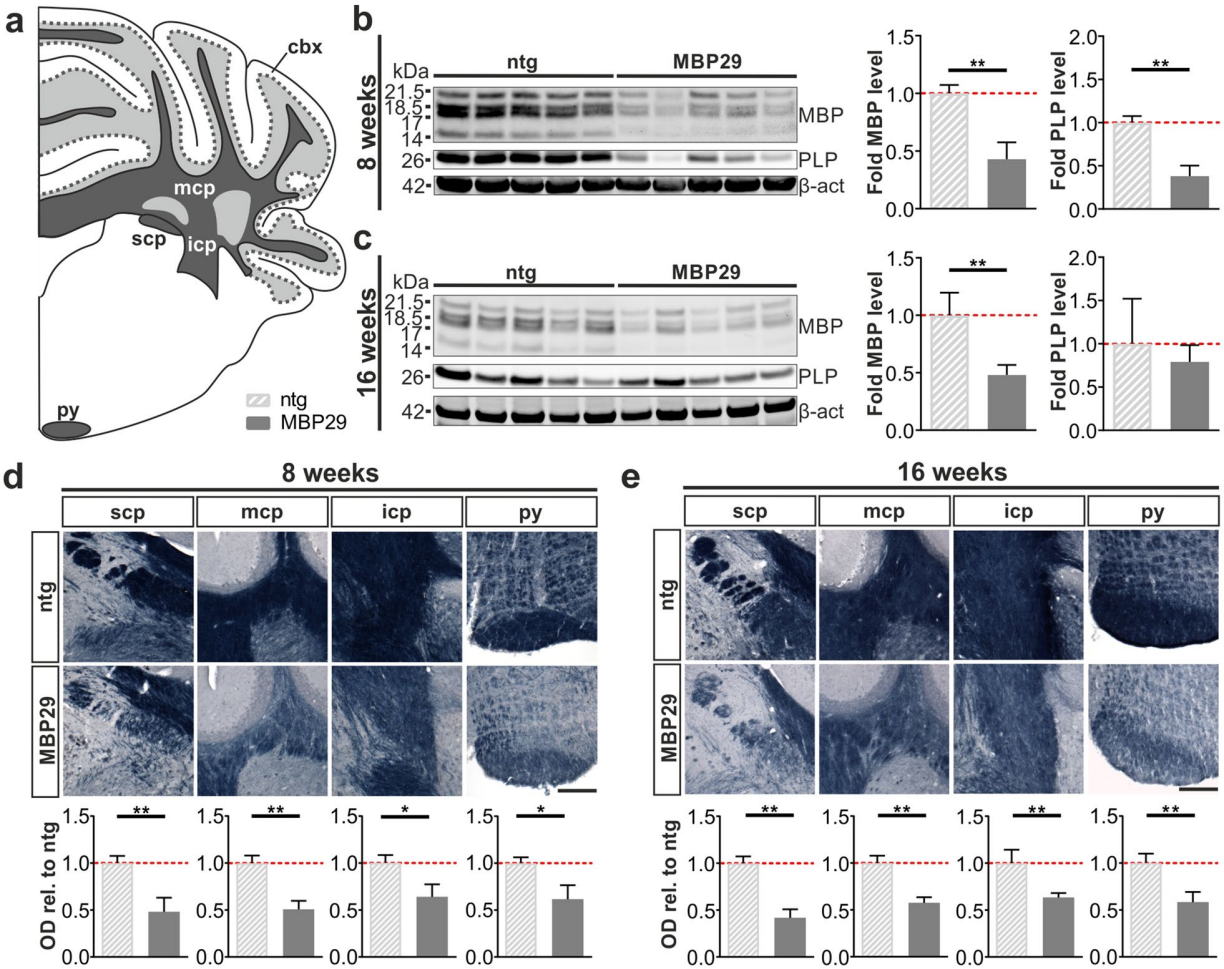
Profound myelin deficit in 8- and 16-week old MBP29-hα-syn (MBP29). **a** Graphical overview illustrating regions of interest within the mouse cerebellum including the superior (scp), middle (mcp), and inferior cerebellar peduncle (icp), as well as the pyramidal tract (py) according to [35]; coordinates: Interaural: − 2.00 mm, Bregma: − 5.79 mm. **b**, **c** Western Blot for myelin basic protein (MBP) and proteolipid protein (PLP). Quantification of MBP and PLP relative to the housekeeper β-actin (β-act) in 8- (**b**) and 16-week-old (**c**) MBP29-hα-syn compared to non-transgenic (ntg) mice (n = 5). **d**, **e** Heidenhain Woelcke staining for myelin. Quantification measured by optical density (OD) in the region of interest normalized to the molecular layer within the cerebellar cortex (cbx) in 8- (**d**) and 16-week-old (**e**) MBP29-hα-syn compared to ntg mice (n = 5–6); scale bar: 20 µm. Data represent mean + standard deviation. Statistical analyses were performed using Mann–Whitney test; *p ≤ 0.05, **p ≤ 0.01

At first, we wanted to examine the expression of human α-syn in the cerebellum of MBP29-hα-syn mice and were indeed able to observe numerous intraoligodendrocytic α-syn accumulations in the cbw of transgenic mice (Additional file 1. Fig. S2a). In addition, we observed the majority of these a-syn accumulations to be phosphorylated (Additional file 1. Fig. S2b). Co-staining of α-syn and TPPP/p25α revealed no differences regarding the localization of TPPP/p25α immunoreactivity (Additional file 1. Fig. S2c). We next performed Western Blot analyses to determine the levels of the myelin-related proteins MBP and PLP in the cerebellum (Fig. 4b, c). We observed a severe reduction of both MBP and PLP, already in 8-week-old transgenic mice by 57% (*p* = 0.008) and 62% (*p* = 0.008), respectively. Additionally, by using the Heidenhain Woelcke staining, we analyzed the myelin lipid content within the cbw, in particular the superior (scp), middle (mcp), and inferior cerebellar peduncle (icp), as well as the pyramidal tract (py) (Fig. 4a). Quantification of the optical density in these regions in MBP29-hα-syn mice (Fig. 4d, e) revealed a loss of myelin ranging from 36 to 52% (*p* < 0.01) already at the prodromal stage of 8 weeks relative to non-transgenic controls. Taken together, these findings confirm the presence of a severe myelin deficit within the cbw including the pyramidal tracts of MBP29-hα-syn mice already at an early disease stage.

**Fig. 5:**
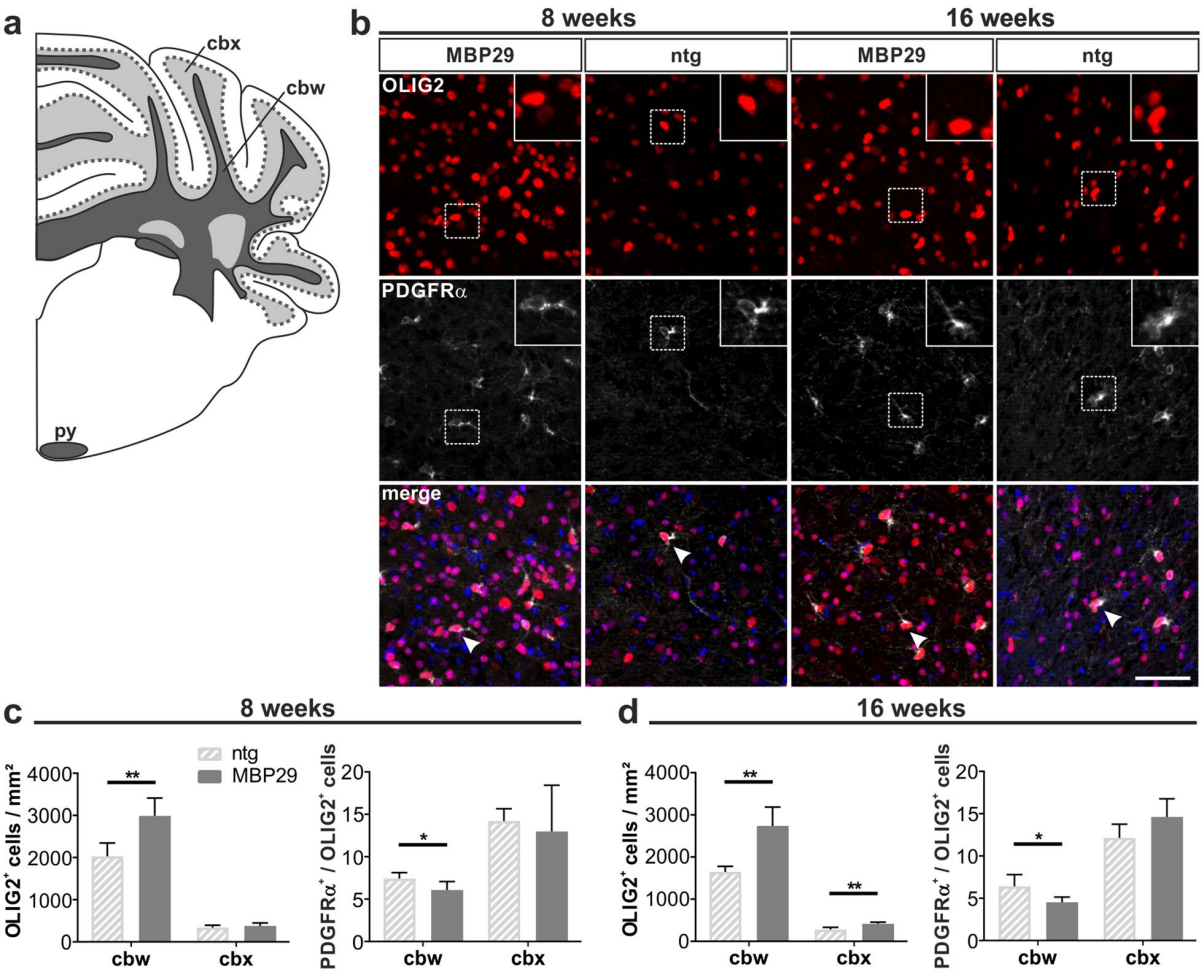
Increased number of OLIG2^+^ oligodendrocytes in 8- and 16-week-old MBP29-hα-syn (MBP29) mice. **a** Graphical overview illustrating regions of interest within the mouse cerebellum including the cerebellar white matter (cbw) and cortex (cbx)  according to  [35]; coordinates: Interaural: -2.00 mm, Bregma: -5.79 mm. **b** Maximum intensity projection images showing OLIG2 (red) as oligodendrocyte-specific marker and PDGFRα (white) as marker for oligodendrocyte precursor cells within the cbw; DAPI^+^ nuclei are shown in blue; scale bar: 50 µm. **c**, **d** The number of OLIG2^+^ cells is increased in the cbw, but not in the cbx in 8- (**c**) and 16-week-old (**d**) MBP29-hα-syn mice (n = 6) compared to non-transgenic (ntg) mice (n = 6). The ratio of oligodendrocyte precursor cells to total oligodendrocyte number are expressed as proportion of PDGFRα to OLIG2 given in percentage. Note the proportional decrease of PDGFRα + oligodendrocyte precursor cells in MBP29-hα-syn mice at 8 and 16 weeks of age. Data represent mean + standard deviation. Statistical analyses were performed using Mann–Whitney test; *p ≤ 0.05, **p ≤ 0.01

**Fig. 6:**
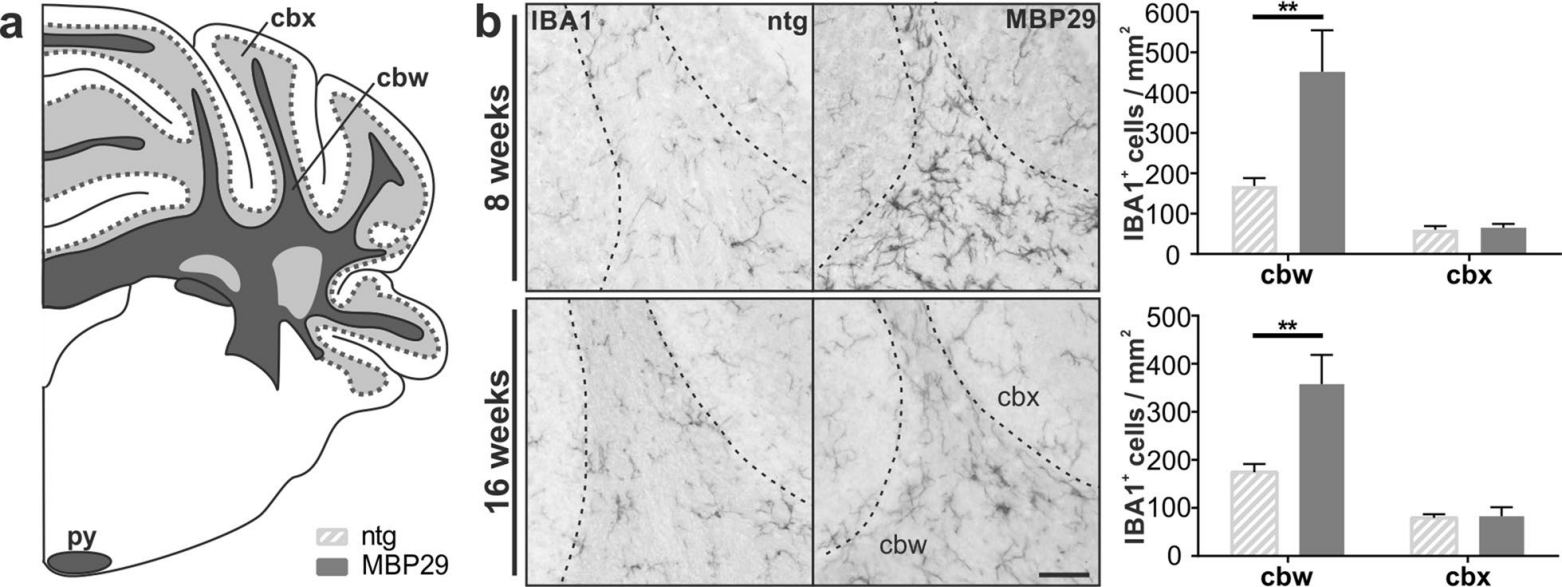
Increased number of IBA1^+^ microglia in cerebellar white matter in MBP29-hα-syn (MBP29) mice at 8 and 16 weeks of age. **a** Graphical overview illustrating regions of interest within the mouse cerebellum including the cerebellar white matter (cbw) and cortex (cbx)  according to [35] ; coordinates: Interaural: -2.00 mm, Bregma: -5.79 mm. **b** The number of IBA1^+^ cells as marker for myeloid cells is significantly increased in the cbw in 8- and 16-week old MBP29-hα-syn (n = 6) compared to non-transgenic (ntg) mice (n = 6). Note the contrast of microgliosis in cbw and cbx. Scale bar: 50 µm. Data represent mean + standard deviation. Statistical analyses were performed using Mann–Whitney test; **p ≤ 0.01

Next, we examined whether the profound myelin deficit in MBP29-hα-syn mice may be linked to a reduced number of oligodendrocytes. Similar to the analysis of the human *post-mortem* tissue (Fig. 2d, e), we quantified the number of OLIG2^+^ cells (Fig. 5). Exceeding our findings of the *post-mortem* examination, the number of OLIG2^+^ cells was increased in the cbw of MBP29-hα-syn mice compared to controls already at an age of 8 weeks (1.5-fold, *p* = 0.002) and remained at this level at 16 weeks of age (1.7-fold, *p* = 0.002). In contrast, the oligodendrocyte number remained at a low level in the cbx of 8-week-old and slightly increased in 16-week-old transgenic mice compared to controls. Size distribution of OLIG2^+^ nuclei revealed no differences between transgenic and control mice (data not shown). To determine whether the increased number of oligodendrocytes is due to an elevated number of immature oligodendrocyte precursor cells, we additionally quantified the number of OLIG2^+^ cells expressing PDGFRα. To our surprise, the proportion of PDGFRα^+^ oligodendrocyte precursor cells in relation to the total number of oligodendrocytes was not elevated, but even slightly reduced in cbw of 8- and 16-week-old MBP29-hα-syn mice (18%, *p* = 0.04 and 29%, *p* = 0.02, respectively). This data strongly implies an oligodendrocytic dysfunction in MBP29-hα-syn mice leading to the observed myelin deficit. Furthermore, examination of the neuroinflammatory pattern in the cbw and cbx of MBP29-hα-syn mice was performed by quantifying the number of IBA1^+^ cells (Fig. 6), a widely used marker for activated and resting myeloid cells. Astonishingly, the density of IBA1^+^ cells was increased by 2.7-fold (*p* = 0.002) in the cbw already at the prodromal stage of 8 weeks and remained at this level at 16 weeks of age (Fig. 6b). In contrast, no difference was observed in the cbx of MBP29-hα-syn mice compared to controls at both time points.

**Fig. 7:**
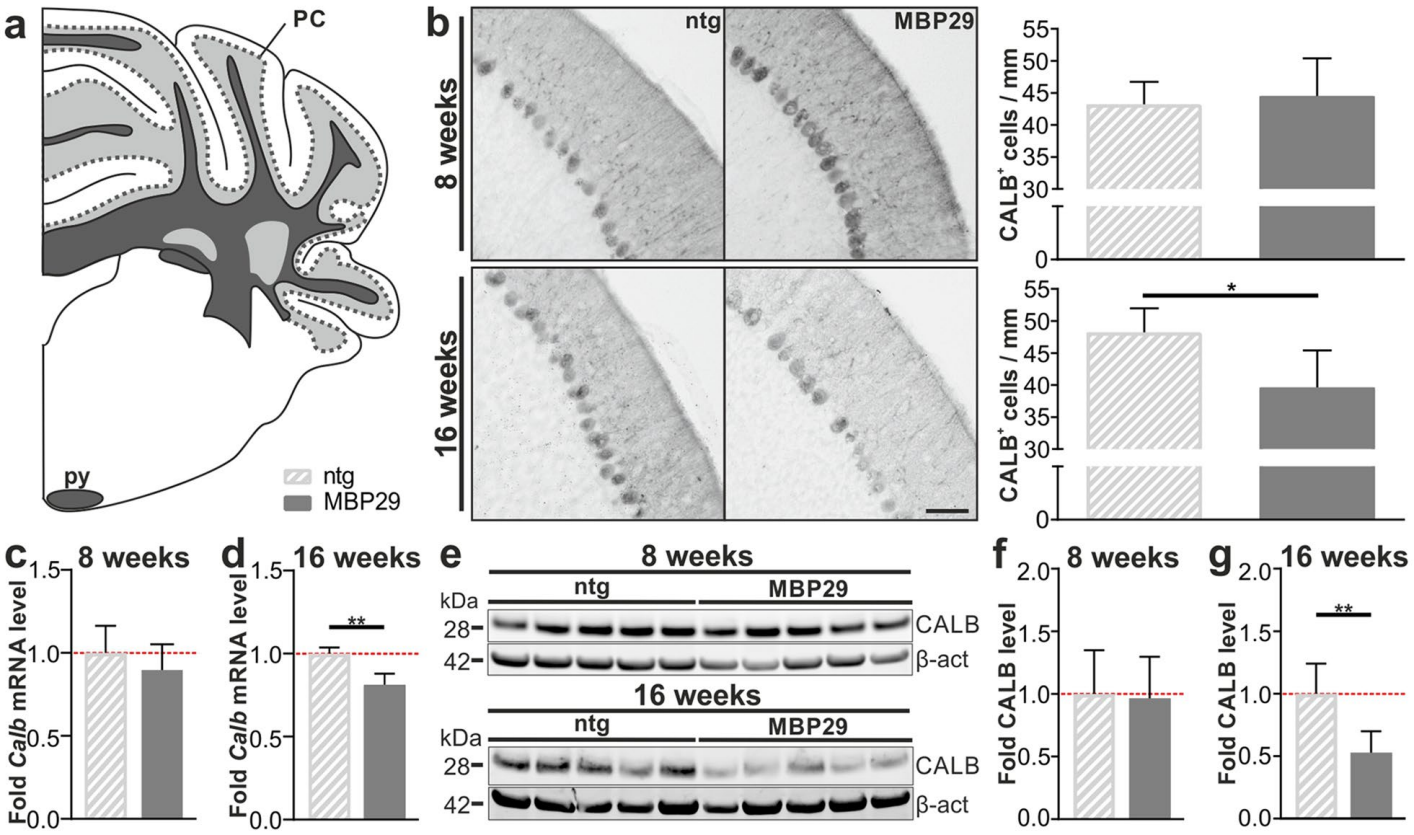
Progressive loss of calbindin^+^ Purkinje cells (PC) and severely decreased expression of CALB in MBP29-hα-syn (MBP29) mice at 16 weeks of age. **a** Graphical overview illustrating regions of interest within the mouse cerebellum including the Purkinje cell monolayer according to [35]; coordinates: Interaural: -2.00 mm, Bregma: -5.79 mm. **b** Calbindin^+^ (CALB) Purkinje cells in 8- and 16-week-old mice (n = 6). Scale bar: 50 µm. **c**, **d** Quantification of *Calb* mRNA expression relative to the housekeepers *ß-actin* and *18S rRNA* expressed as fold change over the mean ntg levels in 8- (**c**) and 16-week-old (**d**) MBP29-hα-syn compared to non-transgenic (ntg) mice (n = 5). **e** Western Blot for CALB and the housekeeper β-actin (β-act) in 8- and 16-week-old mice (n = 5). **f**, **g** Quantification of the protein level of CALB relative to ß-actin in 8- (**f**) and 16-week-old (**g**) MBP29-hα-syn compared to ntg mice. Data represent mean + standard deviation. Statistical analyses were performed using Mann–Whitney test; *p ≤ 0.05, **p ≤ 0.01, ***p ≤ 0.001

Besides analyzing neuroinflammation and myelin levels, we quantified the number of Purkinje cells within the cerebellum. For this purpose, we assessed the number of CALB^+^ cells, a prototypical marker for Purkinje cells, in the cbx of MBP29-hα-syn mice and controls (Fig. 7a, b). While we observed no differences between 8-week-old transgenic mice and controls, the number of CALB^+^ cells was reduced in 16-week-old transgenic mice by 18% (*p* = 0.015). To validate these findings, we additionally analyzed the mRNA (Fig. 7c, d) and protein expression (Fig. 7e–g) of CALB in 8- and 16-week-old MBP29-hα-syn mice and non-transgenic controls. We observed a corresponding decline of CALB expression in 16-week-old transgenic mice only (mRNA by 19%, *p* = 0.008; protein by 47%, *p* = 0.008).

Taken together, our findings imply an early onset of a severe myelin deficit despite an increased number of oligodendrocytes accompanied by an increase of myeloid cells in MBP29-hα-syn mice. In contrast, the severe loss of Purkinje cells and reduction of CALB expression occurs at a rather late stage of the disease likely contributing to the motor phenotype of MBP29-hα-syn mice.

### Changes in gait pattern of MBP29-hα-syn mice

**Fig. 8:**
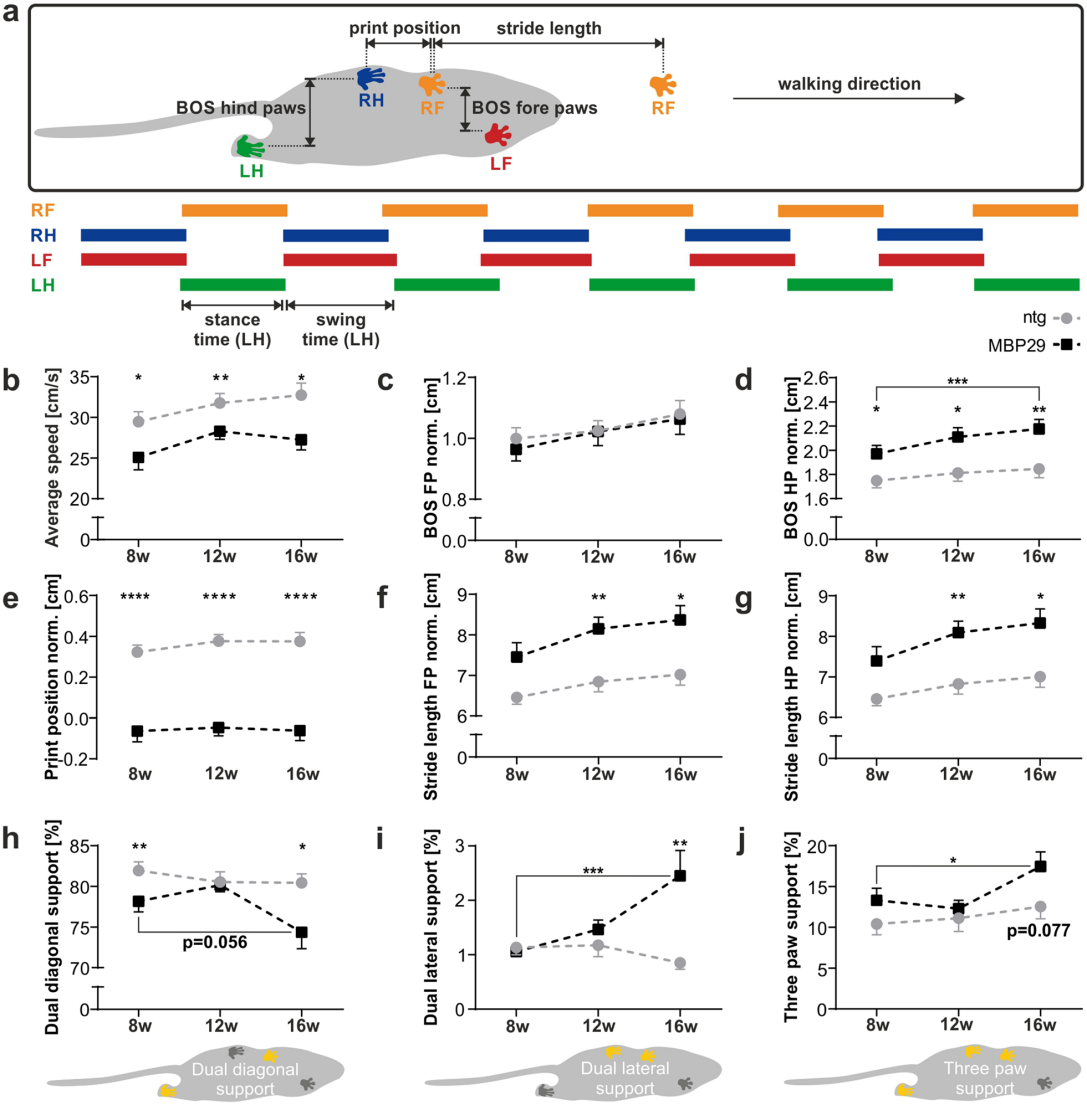
Gait pattern of MBP29-hα-syn mice (MBP29). **a** Graphical overview illustrating gait parameters measured by the Catwalk XT gait analysis system including the print position, base of support (BOS) of fore (FP) and hind paws (HP), stride length, as well as swing and stance time. Abbreviations: Right fore paw (RF), right hind paw (RH), left fore paw (LF), and left hind paw (LH). **b**–**j** Ataxic gait in MBP29-hα-syn (n = 24) compared to non-transgenic (ntg) mice (n = 24) at 8, 12, and 16 weeks of age. Parameters include average speed (**b**), BOS of fore (**c**), and hind paws (**d**), print position (**e**), stride length of fore (**f**) and hind paws (**g**) normalized (norm.) to the mean bodyweight at each time point, as well as the proportion of dual diagonal (**h**), dual lateral (**i**), and three paw support (**j**). Paws highlighted in yellow illustrate the respective paw position i.e. dual diagonal (**h**), dual lateral (**i**), and three paw support (**j**). Data represent mean ± standard error of the mean. Statistical analyses were performed using Mann–Whitney test for comparison of MBP29-hα-syn and ntg mice at each time point, Wilcoxon signed-rank test for comparison of 8- and 16-week-old MBP29-hα-syn mice; *p ≤ 0.05, **p ≤ 0.01, ***p ≤ 0.001, ****p ≤ 0.0001

**Fig. 9:**
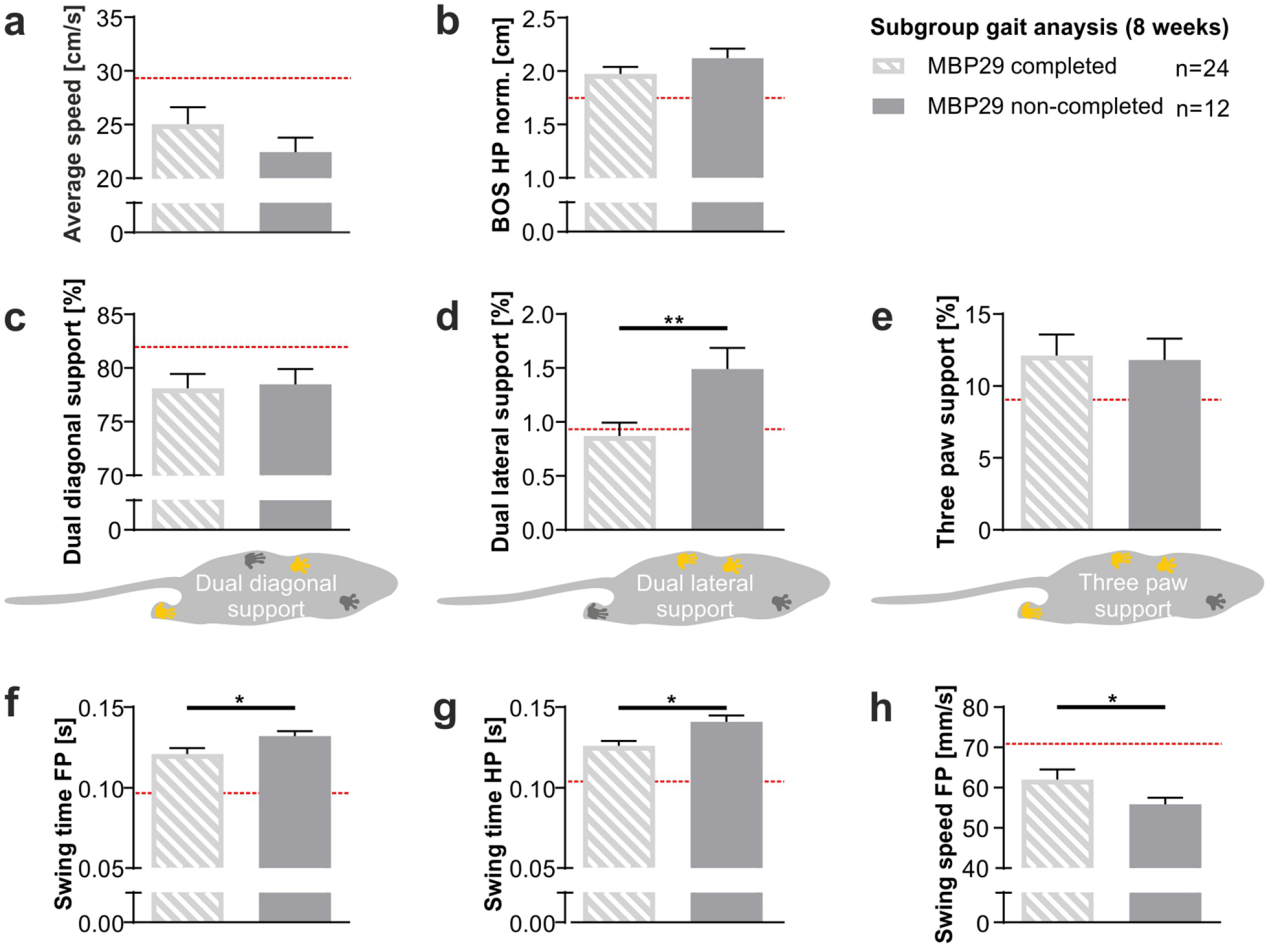
Gait pattern of MBP29-hα-syn mice (MBP29 completed) compared to MBP29-hα-syn mice (MBP29 non-completed) at an age of 8 weeks. **a**–**h** Analysis of gait parameters by the Catwalk XT gait analysis system indicating ataxic gait of MBP29-hα-syn mice with slow disease progression (n = 24) versus early onset MBP29-hα-syn mice (n = 12) at 8 weeks of age. Note that non-transgenic controls (ntg) are presented as red line for graphical comparisons. Parameters include average speed (**a**), base of support (BOS) of hind paws (HP) normalized (norm.) to the mean bodyweight (**b**), the proportion of dual diagonal (**c**), dual lateral (**d**), and three paw support (**e**), as well as swing time of fore paws (FP) (**f**) and hind paws (HP) (**g**), and swing speed of FP (**h**). Paws highlighted in yellow illustrate the respective paw position i.e. dual diagonal (**c**), dual lateral (**d**), and three paw support (**e**). Data represent mean + standard error of the mean. Statistical analyses were performed using Mann–Whitney test; *p ≤ 0.05, **p ≤ 0.01

We characterized the gait of MBP29-hα-syn mice at 8, 12, and 16 weeks of age using the Catwalk XT 10.0 system in order to analyze the impact of cerebellar neuropathology on motor function (Fig. 8, Additional file 1. Table S1). It must be noted that we normalized all parameters expressed in length units to the mean bodyweight at each time point due to mixed-sex cohorts and weight gain during assessment in order to control for confounding effects. First, we discovered a reduced average walking speed by 15% that was already present in 8-week-old MBP29-hα-syn mice compared to controls (*p* = 0.043; Fig. 8b). Notably, transgenic mice showed a slight drop in walking speed from week 12 to 16 compared to controls reflecting the progressive nature of a functional deficit. We detected no differences in paw width (base of support, BOS) between fore paws, whereas width between hind paws was increased already in 8-week-old MBP29-hα-syn mice compared to controls (*p* = 0.035; Fig. 8c, d) indicating a wide-based gait of the hind limbs. The BOS between hind paws increased during disease progression by 17% (*p* = 0.002). Moreover, the print position between fore and hind paw was reduced in transgenic mice compared to controls at each time point by approximately 85% (*p* < 0.001; Fig. 8e). Interestingly, the stride length of both fore and hind paws, was increased by 14–22% in transgenic mice compared to controls at 12 and 16 weeks of age (Fig. 8f, g). It is remarkable that the dual diagonal support, considered as the natural gait pattern in mice, not only decreased in MBP29-hα-syn mice in comparison to controls, especially at 16 weeks of age, by 8% (*p* = 0.011, r = −0.37), but also dropped considerably between 8 and 16 weeks of age (*p* = 0.056, Fig. 8h). As a consequence, dual lateral support (*p* = 0.004) as well as three paw support (*p* ≥ 0.013) increased during disease progression reaching 3.4-fold (*p* = 0.001) and 1.4-fold (*p* = 0.077), respectively, in 16-week-old transgenic mice compared to controls, indicating a pronounced unsteady gait (Fig. 8i, j). Additional parameters with significant alteration in transgenic mice include decreased swing speed, increased swing and stance time, as well as print width and length (Additional file 1. Table S1). Finally, we performed a subgroup analysis at week 8 and compared the gait pattern of MBP29-hα-syn mice that dropped out prior to the last time point at 16 weeks in contrast to the majority of MBP29-hα-syn mice completing all three time points (Fig. 9, Additional file 1. Table S2). While we observed no changes in parameters such as average speed, base of support of hind paws, dual diagonal, and three paw support (Fig. 9a–c, e), we observed an increase of dual lateral support (1.7-fold, *p* = 0.009) in the cohort unable to complete the longitudinal gait analysis (Fig. 9d). In addition, we discovered swing time and swing speed as possible predictors indicating an accelerated disease progression (Fig. 9f–h, Additional file 1. Table S2).

## Discussion

The present study describes a transgenic mouse model mirroring important features of both MSA-C pathology and cerebellar ataxia. In addition to a widespread α-syn pathology, MBP29-hα-syn mice show a profound deficit of myelin and a distinct neuroinflammatory burden in the cbw followed by a substantial loss of Purkinje cells during disease progression. Furthermore, MBP29-hα-syn mice develop an unsteady and wide-based gait as characteristic manifestation of cerebellar ataxia. Finally, functional predictors for an early detrimental course are distinct gait parameters such as dual lateral support.

### MBP29-hα-syn mice present characteristic MSA-C-associated cerebellar pathology

The most prominent hallmark of MSA are GCIs of α-syn, a mandatory criterion for the “definite” diagnosis after death [5]. Based on our results and previous studies, GCIs are most numerous in the cbw of MSA-C patients already at an early stage of disease [1, 8, 9, 36, 37]. Interestingly, we did not identify GCI pathology in the cbx of MSA-C patients confirming previous results [38]. As the expression of human α-syn is controlled by the oligodendrocyte-specific murine MBP promoter in MBP29-hα-syn mice, α-syn is—similar to human *post-mortem* analysis—mainly localized within the white matter of the cerebellum. Interestingly, GCI pathology has been described to correlate with myelinated axons and demyelination within the cerebellum of MSA-C patients [8, 13]. Accordingly, we identified a profound loss of myelin in the cerebellar subcortical white matter of MSA-C patients, and a substantial deficit of myelin lipids in the cerebellar peduncles and pyramidal tract of MBP29-hα-syn mice. We furthermore observed a decline of the myelin-specific proteins MBP and PLP in the cerebellum. One potential explanation for this profound myelin deficit may be the loss of myelin-wrapping oligodendrocytes as an early event of disease. However, our and other previous studies indicate no reduction of the number of oligodendrocytes or only subtle in the putamen and caudate nucleus of MSA patients [11, 18, 21, 39]. Moreover, the density of oligodendrocyte precursor cells was shown to be higher in the cbw of MSA brains [13]. These observations in MSA-P patients or mixed MSA cohorts are consistent with our findings in the cbw of MSA-C patients. Similar to these findings, we identified an increased number of oligodendrocytes in MBP29-hα-syn mice while the proportion of immature PDGFRα^+^ oligodendrocyte precursor cells in relation to the total number of oligodendrocytes slightly decreased. This does not indicate an overall loss but rather a severe dysfunction of mature oligodendrocytes possibly due to an increased intracellular α-syn level. Previous observations support these findings since increased levels of α-syn interfere with oligodendrocyte maturation [24, 40]. Furthermore, the specific loss of oligodendrocytes in the cbx of MSA-C patients in conjunction with the absence of GCI pathology suggests a region-specific heterogeneity of oligodendrocytic subpopulations recently described in multiple sclerosis [41]. Additionally, the pronounced loss of myelin in the cbw is accompanied by increased levels of inflammation in MSA patients [9, 11, 13, 18, 42]. Indeed, we observed an elevated number of myeloid cells particularly in the cbw of MSA-C patients. Consistent with the *post-mortem* analysis, MBP29-hα-syn mice show an increased number of IBA1^+^ myeloid cells in the cbw only. This inflammatory burden is present already at a very early disease stage. As a possible consequence of the myelin deficit and the neuroinflammatory response, degeneration of Purkinje cells has been observed as another important structural feature of MSA-C [17, 18]. Purkinje cells are inhibitory neurons innervating the cbx and regulating motor coordination and balance, the loss of which is furthermore linked to limb and gait ataxia in MSA-C patients [19]. In line with previous studies and our findings in MSA-C *post-mortem* tissue, we observed a loss of CALB^+^ Purkinje cells in MBP29-hα-syn mice. However, this loss was evident in 16-week-old transgenic mice only and was presumably preceded by their dysfunction and the profound myelin loss. It is remarkable that Purkinje cell loss was accompanied by a reduced expression of CALB on mRNA and protein level in 16-week-old MBP29-hα-syn mice. This reduction has also been described in MSA cases and was related to an elevated calcium toxicity as CALB buffers the calcium entry upon stimulation of glutamate receptors [18, 43, 44]. In addition, ataxia has been structurally linked to a loss of CALB rather than the degeneration of Purkinje cells, as Purkinje cell-specific CALB-deficient mice develop deficits in precise motor coordination without affecting Purkinje cell number [45, 46]. Whether decreased levels of CALB in Purkinje cells of MBP29-hα-syn mice is related to the sole loss and/or dysfunction of these cells needs further exploration in future studies.

### MBP29-hα-syn mice present a cerebellar ataxic gait pattern

As a consequence of the underlying olivopontocerebellar atrophy, MSA-C patients show gait and limb ataxia as well as other cerebellar signs [7, 47]. While gait ataxia manifests itself in motor symptoms such as increased postural sway, wide-based stance, and instability during voluntary movements, limb ataxia is characterized by hyper- or hypometric dysmetria (over- and/or undershoot of limb movements), tremor (involuntary oscillation of the limb), and dyssynergia (poor coordination of multi-joint movements) [48, 49]. Based on gait characteristics associated with cerebellar ataxia, we evaluated the gait pattern of MBP29-hα-syn mice. Analogous to MSA-C patients [49], walking speed of MBP29-hα-syn mice is significantly lower, while swing and stance time were higher compared to control mice. Furthermore, the width between hind paws is significantly increased in transgenic mice during disease progression. The widened base of support together with the diminished print position between ipsilateral paws and the increased print length and width indicate considerable walking instability in MBP29-hα-syn mice and may be similar to the wide-based gait of patients with cerebellar ataxia [49]. Interestingly, MBP29-hα-syn mice displayed increased stride length of fore and hind paws. This overshoot of limb movement in transgenic mice may represent hypermetric dysmetria, a typical feature of limb ataxia. Moreover, the dual diagonal support, considered the natural gait pattern in mice, significantly declined in MBP29-hα-syn mice and progressed over time. Furthermore, an extended double limb support phase in MSA-C patients might be linked to the elevated three paw support in MBP29-hα-syn mice [49]. It is remarkable that the proportional increase of dual lateral support in transgenic mice might be comparable to the postural sway detected in patients with cerebellar ataxia. In summary, MBP29-hα-syn mice display characteristic gait features matching important patterns observed in patients with cerebellar ataxia.

It is noteworthy that some gait parameters were significantly altered in MBP29-hα-syn mice already at an early stage of disease without showing an obvious motor phenotype and could serve as early functional predictors. Furthermore, this finding indicates that the myelin deficit and neuroinflammatory response at an early disease stage may affect distinct motor function prior to the overall loss of Purkinje cells. Additionally, print width, dual diagonal, dual lateral, and three paw support deteriorated during disease progression. This raised the question of whether distinct gait parameters may serve as a functional predictor for the course of disease. At 8 weeks of age we therefore compared MBP29-hα-syn mice which completed all three longitudinal gait assessments to MBP29-hα-syn mice that did not complete the longitudinal gait assessment. Notably, dual lateral support was significantly elevated along with an increased swing time and decreased swing speed in animals that did not reach the last time point of gait analysis. These findings indicate that in particular dual lateral support as well as swing time and speed might serve as potential functional predictors for mortality. Moreover, these parameters may represent a functional endpoint for drug discovery in this transgenic mouse model. Nevertheless, we are not able to causally link cerebellar dysfunction and neuropathology to an accelerated disease course. Overall, the present gait analysis data reveal an ataxic gait pattern in MBP29-hα-syn mice possibly linked to the cerebellar neuropathology. However, we cannot exclude the possibility that the neuropathology of forebrain regions may as well contribute to the observed gait pattern and accelerated disease course of MBP29-hα-syn mice.

In conclusion, the present study shows for the first time that transgenic MBP29-hα-syn mice match with important MSA-C pathology and thus may be a powerful model for further investigations of the underlying pathomechanisms in MSA-C. Moreover, this mouse model represents an ideal tool for drug research of the urgently needed symptomatic treatment of cerebellar ataxia and, more importantly, disease-modifying strategies.

## Supplementary Information

**Additional file 1**: **Figure S1: Absence of GCI pathology in the cerebellar cortex of MSA-C patients.** **a:** Graphical overview illustrating regions of interest within the human cerebellum including the granular cell layer (GCL) and molecular layer (ML). **b, c:** Co-staining of OLIG2 (brown) and α-syn (red) within the ML **(b)** and GCL **(c)**. Nuclei were counterstained using haematoxylin (blue). Inserts show OLIG2+ oligodendrocytes. Scale bar: 20 µm. **Figure S2: Expression of human alpha-synuclein (α-syn) predominantly within the cerebellar white matter of MBP29-hα-syn (MBP29) mice**. **a:** Immunofluorescence staining for OLIG2 (magenta) as oligodendrocyte-specific marker and α-syn (green) within the cerebellar white matter (cbw) and cerebellar cortex (cbx) of 8- and 16-week-old MBP29-hα-syn mice compared to ntg mice; scale bar: 50 µm. **b:** Co-staining of phosphorylated α-syn (pS129-α-syn; magenta) and α-syn (green) within the cbw and cbx of 8- and 16-week-old MBP29-hα-syn mice compared to ntg mice; scale bar: 10 µm. **c:** Cellular expression of TPPP/p25α (magenta) and α-syn (green) within cbw and cbx of 8- and 16-week-old MBP29-hα-syn mice compared to ntg mice; scale bar: 10 µm. All images are shown as maximum intensity projection images; DAPI+ nuclei are shown in blue. **Table S1: Changes of gait parameters and bodyweight in MBP29-hα-syn mice (MBP29) vs. non-transgenic controls (ntg). Table S2: Subgroup gait analysis in MBP29-hα-syn (MBP29) mice (completed) compared to MBP29-hα-syn mice (non-completed)**.

## Data Availability

Data will be provided upon request.
