# Supplementary material for: Human alpha-synuclein overexpressing MBP29 mice mimic functional and structural hallmarks of the cerebellar subtype of multiple system atrophy
Source: Acta Neuropathol Commun. 2021 Apr 14;9:68. doi: 10.1186/s40478-021-01166-x (PMC8048356; doi:10.1186/s40478-021-01166-x)
Supplement: Supplementary file 1 — Additional file 1: Figure S1: Absence of GCI pathology in the cerebellar cortex of MSA-C patients. a: Graphical overview illustrating regions of interest within the human cerebellum including the granular cell layer (GCL) and molecular layer (ML). b, c: Co-staining of OLIG2 (brown) and α-syn (red) within the ML (b) and GCL (c). Nuclei were counterstained using haematoxylin (blue). Inserts show OLIG2+ oligodendrocytes. Scale bar: 20 µm. Figure S2: Expression of human alpha-synuclein (α-syn) predominantly within the cerebellar white matter of MBP29-hα-syn (MBP29) mice. a: Immunofluorescence staining for OLIG2 (magenta) as oligodendrocyte-specific marker and α-syn (green) within the cerebellar white matter (cbw) and cerebellar cortex (cbx) of 8- and 16-week-old MBP29-hα-syn mice compared to ntg mice; scale bar: 50 µm. b: Co-staining of phosphorylated α-syn (pS129-α-syn; magenta) and α-syn (green) within the cbw and cbx of 8- and 16-week-old MBP29-hα-syn mice compared to ntg mice; scale bar: 10 µm. c: Cellular expression of TPPP/p25α (magenta) and α-syn (green) within cbw and cbx of 8- and 16-week-old MBP29-hα-syn mice compared to ntg mice; scale bar: 10 µm. All images are shown as maximum intensity projection images; DAPI+ nuclei are shown in blue. Table S1: Changes of gait parameters and bodyweight in MBP29-hα-syn mice (MBP29) vs. non-transgenic controls (ntg). Table S2: Subgroup gait analysis in MBP29-hα-syn (MBP29) mice (completed) compared to MBP29-hα-syn mice (non-completed). [file 40478_2021_1166_MOESM1_ESM.pdf]

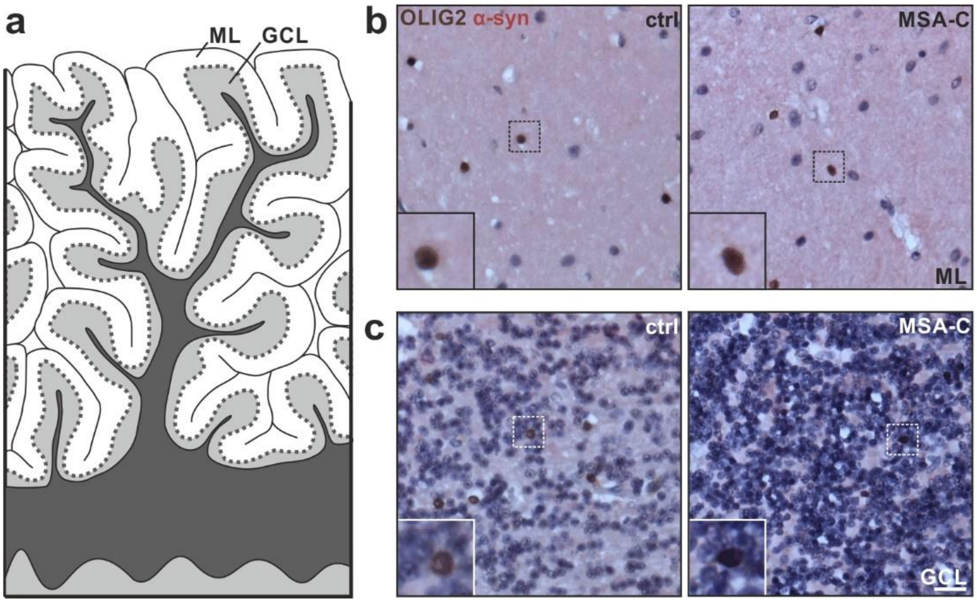

Figure S1: Absence of GCI pathology in the cerebellar cortex of MSA-C patients. a Graphical overview illustrating regions of interest within the human cerebellum including the granular cell layer (GCL) and molecular layer (ML). **b, c** Co-staining of OLIG2 (brown) and α-syn (red) within the ML (**b**) and GCL (**c**). Nuclei were counterstained using haematoxylin (blue). Inserts show OLIG2^{+} oligodendrocytes. Scale bar: 20 µm.

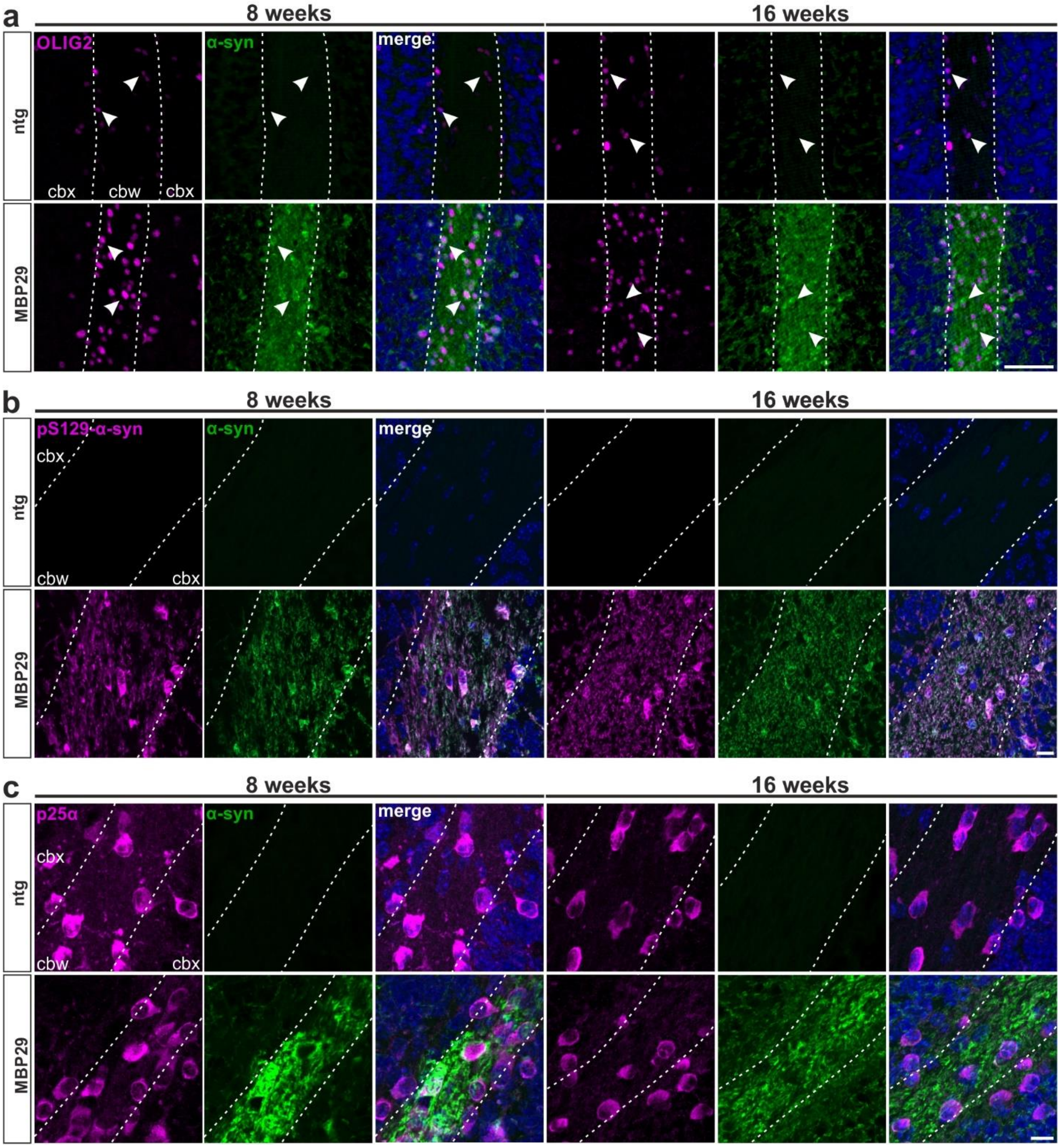

Figure S2: Expression of human alpha-synuclein (α-syn) predominantly within the cerebellar white matter of MBP29-hα-syn (MBP29) mice. **a** Immunofluorescence staining for OLIG2 (magenta) as oligodendrocyte-specific marker and α-syn (green) within the cerebellar white matter (cbw) and cerebellar cortex (cbx) of 8- and 16-weekold MBP29-hα-syn mice compared to ntg mice; scale bar: 50 µm. **b** Co-staining of phosphorylated α-syn (pS129-α-syn; magenta) and α-syn (green) within the cbw and cbx of 8- and 16-week-old MBP29-hα-syn mice compared to ntg mice; scale bar: 10 µm. **c** Cellular expression of TPPP/p25α (magenta) and α-syn (green) within cbw and cbx of 8- and 16-week-old MBP29-hα-syn mice compared to ntg mice; scale bar: 10 µm. All images are shown as maximum intensity projection images; DAPI$^+$ nuclei are shown in blue.

	8 weeks				12 weeks				16 weeks			
	ntg	MBP29			ntg	MBP29			ntg	MBP29		
	mean ± SD	mean ± SD	p	r	mean ± SD	mean ± SD	p	r	mean ± SD	mean ± SD	p	r
**Outcomes**												
Average speed [cm/s]	29.47 ± 5.98	25.09 ± 7.45	0.043	-0.29	31.77 ± 5.68	28.31 ± 4.95	0.009	-0.38	32.74 ± 7.10	27.27 ± 6.24	0.011	-0.37
Swing speed FP [cm/s]	70.99 ± 9.41	61.97 ± 12.40	0.011	-0.37	76.67 ± 8.67	66.48 ± 9.15	0.001	-0.51	77.44 ± 12.09	66.21 ± 9.61	0.003	-0.44
Swing speed HP [cm/s]	63.12 ± 9.89	56.33 ± 10.47	0.017	-0.35	67.88 ± 9.18	61.11 ± 9.35	0.032	-0.31	70.79 ± 11.09	63.34 ± 9.69	0.015	-0.35
Swing time FP [s]	0.10 ± 0.01	0.12 ± 0.02	0.000	-0.67	0.10 ± 0.01	0.12 ± 0.02	0.000	-0.77	0.10 ± 0.01	0.12 ± 0.01	0.000	-0.63
Swing time HP [s]	0.11 ± 0.01	0.13 ± 0.02	0.000	-0.60	0.10 ± 0.01	0.12 ± 0.02	0.000	-0.69	0.10 ± 0.01	0.12 ± 0.02	0.000	-0.57
Stance time FP [s]	0.12 ± 0.02	0.15 ± 0.04	0.002	-0.44	0.12 ± 0.02	0.14 ± 0.02	0.002	-0.46	0.12 ± 0.02	0.15 ± 0.03	0.001	-0.47
Stance time HP [s]	0.11 ± 0.02	0.15 ± 0.05	0.002	-0.45	0.11 ± 0.03	0.13 ± 0.02	0.001	-0.49	0.11 ± 0.02	0.15 ± 0.03	0.000	-0.57
Print position norm. [cm]	0.32 ± 0.17	-0.07 ± 0.26	0.000	-0.69	0.38 ± 0.16	-0.05 ± 0.20	0.000	-0.76	0.38 ± 0.21	-0.06 ± 0.24	0.000	-0.75
Stride length FP norm. [cm]	6.46 ± 0.84	7.46 ± 1.70	0.061	-0.27	6.84 ± 1.18	8.15 ± 1.39	0.003	-0.44	7.02 ± 1.27	8.37 ± 1.74	0.013	-0.36
Stride length HP norm. [cm]	6.46 ± 0.84	7.39 ± 1.74	0.087	-0.25	6.82 ± 1.19	8.09 ± 1.38	0.002	-0.45	7.01 ± 1.27	8.33 ± 1.70	0.014	-0.36
Print width FP norm. [cm]	0.84 ± 0.08	0.93 ± 0.15	0.034	-0.31	0.87 ± 0.09	0.98 ± 0.14	0.006	-0.40	0.88 ± 0.13	0.99 ± 0.15	0.022	-0.34
Print width HP norm. [cm]	0.80 ± 0.11	0.90 ± 0.13	0.015	-0.35	0.86 ± 0.11	0.98 ± 0.12	0.002	-0.46	0.90 ± 0.18	1.05 ± 0.15	0.007	-0.39
Print length FP norm. [cm]	0.46 ± 0.06	0.53 ± 0.08	0.004	-0.42	0.51 ± 0.08	0.58 ± 0.09	0.005	-0.41	0.50 ± 0.10	0.59 ± 0.11	0.018	-0.35
Print length HP norm. [cm]	0.95 ± 0.13	1.11 ± 0.17	0.002	-0.44	1.00 ± 0.16	1.18 ± 0.17	0.001	-0.48	1.02 ± 0.22	1.24 ± 0.21	0.002	-0.46
BOS FP norm. [cm]	1.00 ± 0.17	0.96 ± 0.19	0.458	-0.11	1.02 ± 0.16	1.02 ± 0.23	0.932	-0.01	1.08 ± 0.21	1.06 ± 0.25	1.000	0.00
BOS HP norm. [cm]	1.75 ± 0.29	1.97 ± 0.35	0.035	-0.30	1.81 ± 0.33	2.11 ± 0.38	0.020	-0.34	1.85 ± 0.35	2.18 ± 0.38	0.005	-0.41
Regularity Index [%]	98.78 ± 1.16	98.81 ± 1.44	0.706	-0.05	98.92 ± 0.77	98.46 ± 1.36	0.529	-0.09	98.58 ± 1.13	98.44 ± 1.49	0.864	-0.03
Zero support [%]	0.15 ± 0.35	0.29 ± 0.68	0.542	-0.09	0.12 ± 0.24	0.28 ± 0.72	0.529	-0.09	0.19 ± 0.47	0.43 ± 1.30	0.293	-0.15
Single support [%]	4.08 ± 3.70	3.94 ± 4.90	0.370	-0.13	4.68 ± 4.63	4.35 ± 4.28	0.983	0.00	3.85 ± 4.49	3.74 ± 4.65	0.898	-0.02
Dual diagonal support [%]	81.95 ± 5.30	78.15 ± 6.35	0.009	-0.38	80.55 ± 6.00	80.17 ± 4.01	0.233	-0.17	80.44 ± 5.29	74.35 ± 9.86	0.011	-0.37
Dual lateral support [%]	0.95 ± 0.67	0.87 ± 0.57	0.578	-0.08	0.99 ± 1.01	1.28 ± 0.85	0.087	-0.25	0.67 ± 0.58	2.27 ± 2.28	0.001	-0.49
Dual girdle support [%]	1.74 ± 0.83	1.98 ± 1.44	0.951	-0.01	1.82 ± 1.57	1.70 ± 1.15	1.000	0.00	1.35 ± 0.90	1.43 ± 1.76	0.489	-0.10
Three paw support [%]	9.19 ± 6.41	12.10 ± 7.25	0.103	-0.24	9.90 ± 7.73	11.09 ± 4.93	0.142	-0.21	11.35 ± 7.24	16.26 ± 8.65	0.077	-0.26
Four paw support [%]	1.30 ± 1.38	1.83 ± 2.65	0.549	-0.09	1.93 ± 2.44	1.13 ± 1.29	0.509	-0.10	2.15 ± 2.14	1.53 ± 1.46	0.416	-0.12
Bodyweight	22.76 ± 3.33	21.10 ± 3.92	0.081	-0.25	26.34 ± 4.80	23.52 ± 4.29	0.063	-0.27	28.55 ± 6.12	24.90 ± 5.03	0.015	-0.36

Table S1: Changes of gait parameters and bodyweight in MBP29-hα-syn mice (MBP29) vs. non-transgenic controls (ntg). Changes of gait parameters in MBP29-hα-syn mice (MBP29) vs. non-transgenic controls (ntg) at an age of 8, 12, and 16 weeks using Catwalk XT gait analysis system. Data represent mean ± standard deviation. P-values were analyzed using Mann-Whitney-U test. Effect size r was calculated according to Rosenthal 1991 [34]. Note that significance ($p \leq 0.05$) and medium to large effect size ($|r| \geq 0.3$) are highlighted in gray.

	8 weeks							
	MBP29 completed			MBP29 non-completed				
	mean	±	SD	mean	±	SD	p	r
**Outcomes**								
Average speed [cm/s]	25.09	±	7.45	22.43	±	4.72	0.383	-0.15
Swing time FP [s]	0.12	±	0.02	0.13	±	0.01	0.013	-0.41
Swing time HP [s]	0.13	±	0.02	0.14	±	0.01	0.005	-0.47
Stance time FP [s]	0.15	±	0.04	0.17	±	0.03	0.100	-0.27
Stance time HP [s]	0.15	±	0.05	0.15	±	0.03	0.365	-0.15
Swing speed FP [cm/s]	61.97	±	12.40	55.88	±	5.57	0.044	-0.34
Swing speed HP [cm/s]	56.33	±	10.47	49.55	±	6.68	0.070	-0.30
Print position norm. [cm]	-0.07	±	0.26	-0.09	±	0.24	1.000	0.00
Stride length FP norm. [cm]	7.46	±	1.70	7.66	±	1.06	0.663	-0.07
Stride length HP norm. [cm]	7.39	±	1.74	7.61	±	1.08	0.638	-0.08
Print width FP norm. [cm]	0.93	±	0.15	0.92	±	0.10	0.763	-0.05
Print width HP norm. [cm]	0.90	±	0.13	0.92	±	0.09	0.663	-0.07
Print length FP norm. [cm]	0.53	±	0.08	0.54	±	0.07	0.615	-0.08
Print length HP norm. [cm]	1.11	±	0.17	1.14	±	0.13	0.568	-0.10
BOS FP norm. [cm]	0.96	±	0.19	1.10	±	0.19	0.052	-0.32
BOS HP norm. [cm]	1.97	±	0.35	2.12	±	0.31	0.202	-0.21
Regularity Index [%]	98.81	±	1.44	99.03	±	0.70	0.697	-0.07
Zero support [%]	0.29	±	0.68	0.01	±	0.03	0.122	-0.26
Single support [%]	3.94	±	4.90	4.29	±	2.63	0.208	0.21
Dual diagonal support [%]	78.15	±	6.35	78.48	±	4.96	0.840	-0.03
Dual lateral support [%]	0.87	±	0.57	1.49	±	0.68	0.009	-0.44
Dual girdle support [%]	1.98	±	1.44	2.13	±	1.37	0.591	-0.09
Three paw support [%]	12.10	±	7.25	11.80	±	5.14	0.920	-0.02
Four paw support [%]	1.83	±	1.20	1.30	±	4.10	0.775	-0.05
Bodyweight	21.10	±	3.92	20.18	±	2.33	0.880	-0.03

Table S2: Subgroup gait analysis in MBP29-hα-syn (MBP29) mice (completed) compared to MBP29-hα-syn mice (non-completed). Subgroup analysis shows changes of gait parameters in MBP29-hα-syn mice (completed) with slow disease progression versus MBP29-hα-syn mice (non-completed) with a rather rapid disease progression at an age of 8 weeks using Catwalk XT gait analysis system. Note significant changes in swing time, swing speed, and dual lateral support in non-completed MBP29-hα-syn mice compared to MBP29-hα-syn mice that underwent three gait analyses. Data represent mean ± standard deviation of 24 MBP29-hα-syn mice (completed) and 12 MBP29-hα-syn (non-completed) mice. P-values were analyzed using Mann-Whitney test. Effect size r was calculated according to Rosenthal 1991 [34]. Note that significance ($p \leq 0.05$) and medium to large effect size ($|r| \geq 0.3$) are highlighted in gray.
